# Rheological Modeling of Metallic Oxide Nanoparticles Containing Non-Newtonian Nanofluids and Potential Investigation of Heat and Mass Flow Characteristics

**DOI:** 10.3390/nano12071237

**Published:** 2022-04-06

**Authors:** Muhammad Rizwan, Mohsan Hassan, Oluwole Daniel Makinde, Muhammad Mubashir Bhatti, Marin Marin

**Affiliations:** 1 Department of Mathematics, COMSATS University Islamabad, Lahore Campus, Lahore 54000, Pakistan; m.rizwan7571@gmail.com (M.R.); mohsan.hassan@cuilahore.edu.pk (M.H.); 2 Faculty of Military Science, Stellenbosch University, Private Bag X2, Saldanha 7395, South Africa; makinded@gmail.com; 3 College of Mathematics and Systems Science, Shandong University of Science and Technology, Qingdao 266590, China; 4 Department of Mathematics and Computer Science, Transilvania University of Brasov, 500036 Brasov, Romania; m.marin@unitbv.ro

**Keywords:** non-Newtonian nanofluids, mathematical modeling based on experimental data, heat-flow characteristics, power-law fluid model

## Abstract

Nanofluids have great potential due to their improved properties that make them useful for addressing various industrial and engineering problems. In order to use nanofluids on an industrial scale, it is first important to discuss their rheological behavior in relation to heat transfer aspects. In the current study, the flow characteristics of nanofluids are discussed using a mathematical model that is developed by fundamental laws and experimental data. The data are collected in the form of viscosity versus shear rate for different homogeneous ethylene glycol- (EG) based nanofluids, which are synthesized by dispersing 5–20% nanoparticle concentrations of SiO_2_, MgO, and TiO_2_ with diameters of (20–30 nm, 60–70 nm), (20 nm, 40 nm), and (30 nm, 50 nm), respectively. The data are fitted into a rheological power-law model and further used to govern equations of a physical problem. The problem is simplified into ordinary differential equations by using a boundary layer and similarity transformations and then solved through the numerical Runge–Kutta (RK) method. The obtained results in the form of velocity and temperature profiles at different nanoparticle concentrations and diameters are displayed graphically for discussion. Furthermore, displacement and momentum thicknesses are computed numerically to explain boundary-layer growth. The results show that the velocity profile is reduced and the temperature profile is raised by increasing the nanoparticle concentration. Conversely, the velocity profile is increased and the temperature profile is decreased by increasing the nanoparticle diameter. The results of the present investigation regarding heat and mass flow behavior will help engineers design equipment and improve the efficacy and economy of the overall process in the industry.

## 1. Introduction

Nanofluids are advanced classes of heat-transfer fluids designed by diffusing nanometer-scale particles of metal, metal oxide, carbon nanotubes, nitride, carbide, and compound materials in traditional base fluids such as ethylene glycol (EG), water, oils, etc. [1,2,3,4]. As a consequence of the improved properties related to heat transfer and chemical stability [5,6,7,8], nanofluids have become immensely attractive and demonstrate several potential applications in many fields relating to solar collection, transportation, the energy industry, refrigeration, the cooling process, chemistry, biomedicine, and the environment [8,9,10,11,12,13,14,15].

Many studies are reported in the literature on the different properties of nanofluids, such as density, thermal conductivity, viscosity, specific heat, etc. [16,17,18,19,20]. However, it is observed that the most critical properties of nanofluids are their rheological properties. For example, based on the different types of base fluids, the nanofluids containing MWCNTs exhibit both Newtonian and non-Newtonian behavior. The composition of MWCNTs with water, resin, and oil displays non-Newtonian behavior [21,22,23,24,25,26,27,28,29,30]. Normally, nanofluids consisting of MWCNTs reveal Newtonian behavior at low volume fractions and non-Newtonian behavior at high volume fractions [21,22]. In the case of TiO_2_-water nanofluids, the fluids mostly exhibit non-Newtonian behavior [31,32,33,34]. Contrarily, Bobbo et al. [35] and Penkavova et al. [36] reported that, for all compositions, TiO_2_/water nanofluids displayed Newtonian behavior. In the case of SiO_2_, the nanofluids with different base fluids showed behavior that was close to Newtonian behavior [37,38,39,40,41,42]. In short, the nanofluids exhibited non-Newtonian behavior [43,44,45,46,47] in many cases, whereas few showed Newtonian behavior [35,36,48].

Non-Newtonian nanofluids that were synthesized by water or EG revealed shear-thinning behavior [43,44,45,46,47]. Shear-thinning fluid is a kind of non-Newtonian fluid wherein viscosity declines by the rise of the shear rate. There are several mathematical models in the literature used to investigate the rheological behavior of such fluids. In the list of models, the power-law model demonstrates the relation between viscosity and shear rate. It is very popular in various disciplines, such as the biosciences and food and processing reservoir engineering [6,7,8,9,10,11,12,13,14]. Specifically, it is widely used in fluid flow problems under different conditions, and it is even used as a working principle in different kinds of rheometers.

In previous numerical studies, a deficiency was found in the theoretical models that can guess the exact behavior of nanofluids. The researchers used the Newtonian model for homogeneous nanofluids, which does not apply to the experimental behavior of all cases. No one study is available where homogeneous nanofluids deal with the non-Newtonian model. In the current study, we used a non-Newtonian power-law fluid model according to the trend of an experimental study. The parameters of the model are expressed as the function of nanoparticles, and we developed new mathematical relations to these parameters. By using these relations that govern equations, the physical problem became complex because every parameter is a function of the volume fraction, and solving this problem is not easy.

In this study, our goal is to investigate the flow behavior of non-Newtonian nanofluids, which are synthesized by the dispersion of metallic oxides in EG. For the flow, wedge shape is adopted as the geometry for our problem, which is favorable for accelerating or decelerating the fluids. The mathematical problem for flow is developed by the fundamental equations of fluid mechanics and modifies its parameters in view of the experimental evidence. The results from these equations are obtained in the form of velocity and temperature profiles and displayed in graphical form for discussion.

## 2. Nanofluid Modeling

**Figure 1:**
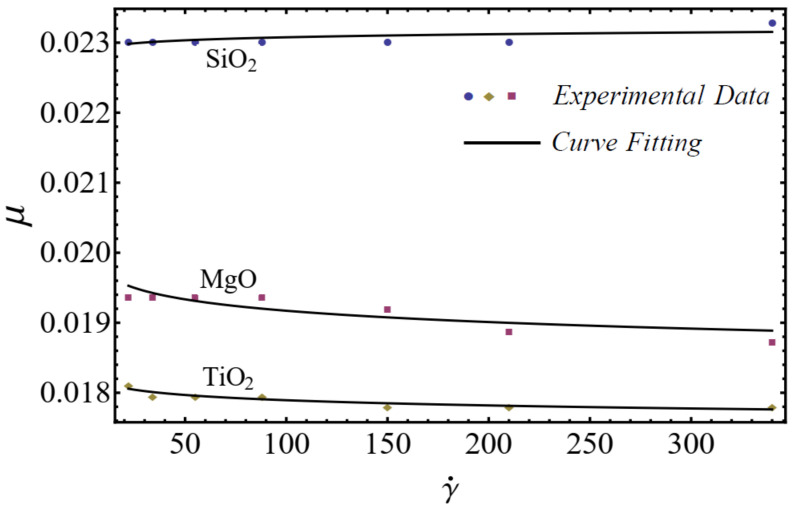
Experimental and mathematical rheological behavior of SiO_2_-EG, MgO-EG, and TiO_2_-EG nanofluids at 5% particle concentration.

**Figure 2:**
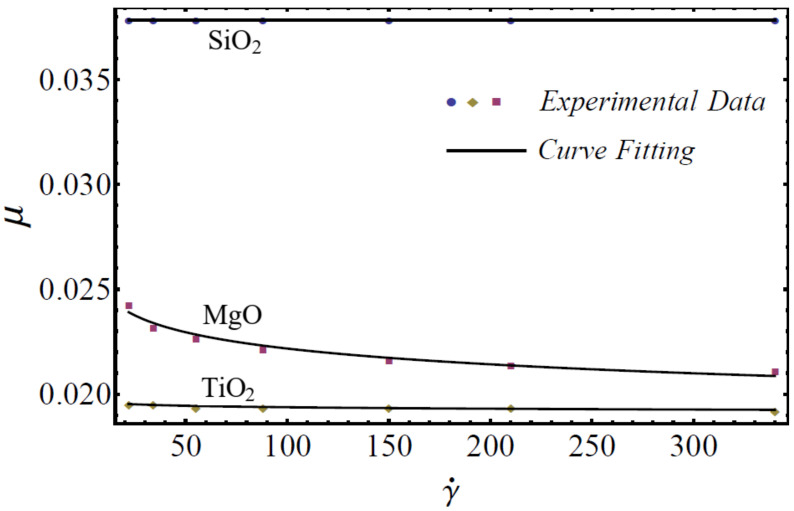
Experimental and mathematical rheological behavior of SiO_2_-EG, MgO-EG, and TiO_2_-EG nanofluids at 10% particle concentration.

**Figure 3:**
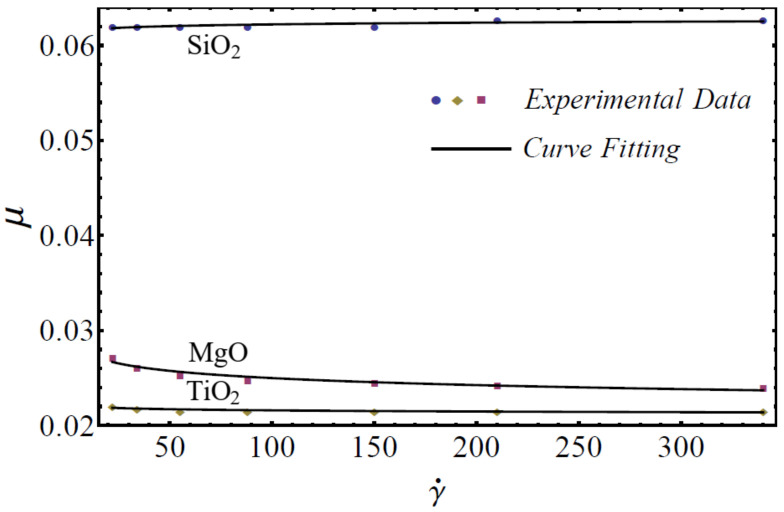
Experimental and mathematical rheological behavior of SiO_2_-EG, MgO-EG, and TiO_2_-EG nanofluids at 15% particle concentration.

In this section, the mathematical models of physical properties are developed by using experimental data for three homogenous nanofluids: SiO_2_-EG, MgO-EG, and TiO_2_-EG. The experimental data for nanofluids, which contain 5%, 10%, 15%, and 20% nanoparticle concentrations and (20–30 nm, 60–70 nm), (30 nm, 50 nm), and (20 nm, 40 nm) nanoparticle diameters, respectively, are collected for the study [1]. We have chosen the nanoparticles of materials SiO_2_, MgO, and TiO_2_ for the nanofluid because these materials are used in manufacturing on a large scale at industry level. EG is used as a base fluid because it can be utilized within sufficiently large temperature ranges. For rheological behavior, the power-law equation is applied for nanofluid modeling in the formation of the viscosity–shear rate relationship as follows [49]:

(1) $$\mu \left(\dot{\gamma }\right)=\mu_{nf}{\dot{\gamma }}^{n-1}$$

where $\mu_{nf}$ is named as the consistency coefficient and n is the power index, which is justifiable according to the Newtonian or non-Newtonian behavior of fluids. When n=1, the fluid exhibits Newtonian behavior, and the fluid shows shear-thinning behavior when n<1. The rheological behavior of said nanofluids at different nanoparticle concentrations is displayed in Figure 1, Figure 2 and Figure 3.

The curve-fitting technique is used to calculate the values of the two empirical parameters $\left(\mu_{nf}\right)$ and $\left(n\right)$ of Equation (1) for different nanofluids at different nanoparticle concentrations. For the curve fitting, we used the FindFit package from Mathematica, which is used for fitting in non-linear models. Afterward, these parameters are expressed in a second-order polynomial of the nanoparticle concentration ϕ. The equation of the power-law index and consistency index is defined as follows:

(2) $$\left.\begin{array}{c} n=1+A_{1}\phi +A_{2}\phi^{2}\\ \mu_{nf}=\left(1+B_{1}\phi +B_{2}\phi^{2}\right)\mu_{bf} \end{array}\right\}$$

where $A_{1}$, $A_{2}$, $B_{1}$, and $B_{2}$ are constants calculated by using the curve-fitting technique in Table 1.

**Figure 4:**
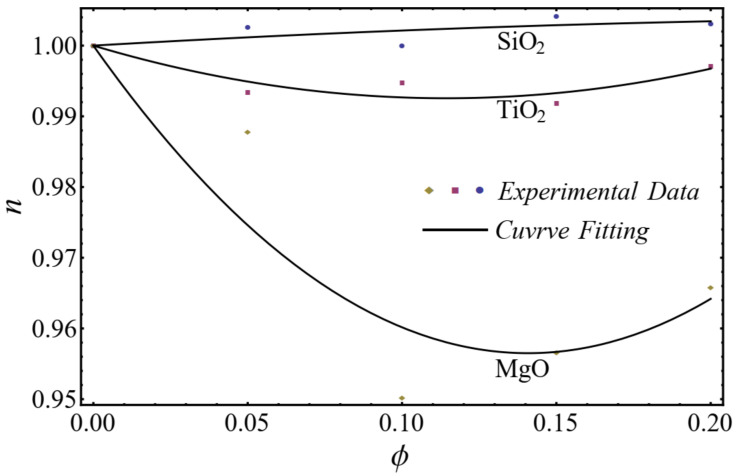
Curve fitting with experimental data for power-law index by using Equation (2).

**Figure 5:**
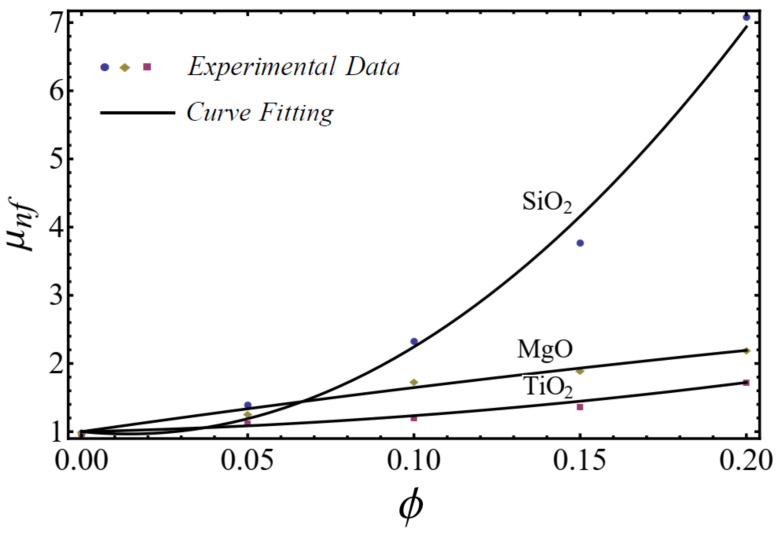
Curve fitting with experimental data for consistency index by using Equation (2).

A good agreement is found between the calculated values and the polynomial equations, as seen in Figure 4 and Figure 5.

Therefore, the viscosity model of Equation (1) is written as

(3) $$\frac{\mu }{\mu_{bf}}=\left(1+B_{1}\phi +B_{2}\phi^{2}\right){\dot{\gamma }}^{1+A_{1}\phi +A_{2}\phi^{2}}$$

The co-relation models for other physical properties, such as heat capacitance ${\left(\rho C_{p}\right)}_{nf}$, effective density $\left(\rho_{nf}\right)$, and thermal conductivity $\left(k_{nf}\right)$, are defined by [50].

(4) $$\rho_{nf}=\phi \;\rho_{np}+\left(1-\phi \;\right)\rho_{bf}$$

(5) $${\left(\rho C_{p}\right)}_{nf}=\phi {\left(\rho C_{p}\right)}_{np}+\left(1-\phi \;\right)\rho_{bf}$$

(6) $$k_{nf}=\frac{k_{pe}+2k_{bf}+2\left(k_{pe}-k_{bf}\right)\;{\left(1+\beta^{*}\right)}^{3}\phi }{k_{pe}+2k_{bf}-\left(k_{pe}-k_{bf}\right)\;{\left(1+\beta^{*}\right)}^{3}\phi }k_{bf}$$

In the above equations, the thermal conductivity of nanoparticle $k_{npl}$ with a layer around the particle is defined by

(7) $$k_{pe}=\frac{\left[2\left(1-\gamma \right)+{\left(1+\beta^{*}\right)}^{3}\left(1+2\gamma^{*}\right)\right]\;\gamma^{*}}{-\left(1-\gamma^{*}\right)+{\left(1+\beta^{*}\right)}^{3}\left(1+2\gamma^{*}\right)}k_{np}$$

where $\gamma^{*}=k_{layer}/k_{np}$ is the ratio of the layers of the nanoparticle to the thermal conductivities of the nanoparticle and $\beta^{*}=h_{layer}/R_{p}$ is the ratio of the layer’s height to the radius of the nanoparticle.

## 3. Flow Modeling

**Figure 6:**
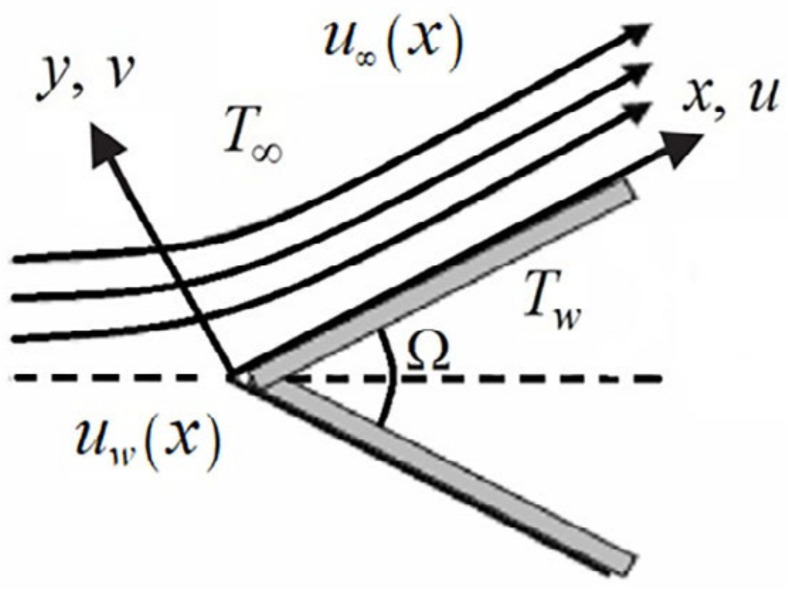
Structure of the flow.

We considered the boundary-layer fluid flow of nanofluids over a moving wedge, which has incompressible and steady-state properties. The wedge is moved with the velocity $u_{w}\left(x\right)=cx^{m}$, whereas $u_{\infty }\left(x\right)=bx^{m}$ is the velocity of the nanofluid over an inviscid region, as shown in Figure 6.

On the basis of the results in Figure 1, Figure 2 and Figure 3, the power-law model is used in momentum Equation (9). Under the boundary-layer approximation, the continuity, momentum, and energy equations are written as [51,52]

(8) $$u_{x}+v_{y}=0$$

(9) $$\rho_{nf}\left(u\;u_{x}+v\;u_{y}\right)=-\partial_{x}\left(p_{e}\right)+\mu_{nf}\;\partial_{y}\left({\left|u_{y}\right|}^{n-1}u_{y}\right)$$

(10) $${\left(\rho C_{p}\right)}_{nf}\left(u\;T_{x}+v\;T_{y}\right)=k_{nf}\;\partial_{y}(T_{y})$$

subjected to the boundary conditions

(11) $$\left.\begin{array}{l} u\left(x,0\right)=-u_{w}\left(x\right),\;\;v\left(x,0\right)=0,\;T\left(x,0\right)=T_{w}\;\;\\ u\left(x,\infty \right)=u_{\infty }\left(x\right),\;T\left(x,\infty \right)=T_{\infty } \end{array}\right\}$$

In the above equations, u and v are the velocity component parts in x and y directions. $T_{w}$ is the temperature on the wedge’s surface, and $T_{\infty }$ is the temperature away from the surface $\left(T_{w}>T_{\infty }\right)$.

The relationship between the Falkner–Skan power-law parameter $\left(m\right)$ and the wedge’s angle β=Ω/π is stated as

(12) $$m=\frac{\beta }{2-\beta }$$

For simplicity, we introduced the similarity transformations as follows:

(13) η=yxRex11+n, ψ=u∞xRex−11+nfη, θη=T−T∞Tw−T∞,  u=∂ψ∂x,v=−∂ψ∂y

Substituting Equation (14) into Equations (9)–(12), we obtain the following equations:

(14) $$n\frac{\mu_{nf}}{\mu_{bf}}\left({\left|f\prime \prime \right|}^{n-1}f\prime \prime \prime \right)+\frac{\rho_{nf}}{\rho_{bf}}\left[\left(\frac{m\left(2n-1\right)+1}{\left(n+1\right)\left(m+1\right)}\right)ff\prime \prime \frac{m}{m+1}f\prime^{2}\right]+\frac{m}{m+1}=0$$

(15) $$\frac{k_{nf}}{k_{bf}}\theta \prime \prime +\mathrm{Pr}\frac{{\left(\rho C_{p}\right)}_{nf}}{{\left(\rho C_{p}\right)}_{bf}}\left(\frac{m\left(2n-1\right)+1}{\left(n+1\right)\left(m+1\right)}\right)f\theta \prime =0$$

with the boundary conditions

(16) $$\left.\begin{array}{c} f\left(0\right)=\;0,\;f\prime \left(0\right)=-\lambda ,\;\theta \left(0\right)=1,\;\\ \theta \left(\infty \right)=0,\;f\prime \left(\infty \right)=1, \end{array}\right\}$$

Here, the modified Prandtl number is Prx=ρbfcpbfu∞xkbfRex2n−1, and the Reynold number is Rex=(m+1)u∞2−nxnνbf and $\lambda =\frac{u_{w}}{u_{\infty }}$ velocities ratio.

## 4. Physical Parameters

### 4.1. Displacement Thickness

Displacement thickness is recognized as the vertical distance that is produced by the absent mass flow rate due to the boundary-layer phenomena. The expression for displacement thickness is written as

(17) $$\delta^{*}=\int_{0}^{\infty }\left(1-\frac{u}{u_{\infty }}\right)dy$$

Dimensionless displacement thickness is composed as

(18) δ∗=xRex−11+n∫0∞1−f′dη

### 4.2. Momentum Thickness

Momentum thickness is the height of an imaginary stream, which is transmitted by the loss of the momentum flow rate due to the boundary-layer phenomena and is described mathematically as

(19) $$\delta^{**}=\int_{0}^{\infty }\frac{u}{u_{\infty }}\left(1-\frac{u}{u_{\infty }}\right)dy$$

Dimensionless momentum thickness is illustrated as

(20) δ∗∗=xRex−11+n∫0∞f′1−f′dη

### 4.3. Skin Friction

The skin-friction coefficient is a dimensionless parameter that represents the shear stress at the wall. It is expressed as

(21) $$C_{f}=\frac{\tau_{w}}{\frac{1}{2}\rho_{bf}u_{\infty }{}^{2}}$$

The wall shear stress $\tau_{w}$ can be written as

(22) $$\tau_{w}=\mu {\left.u_{y}\right|}_{y=0}$$

After applying transformation Equation (13), we receive

(23) Cf=2m+1Rex−11+nμnfμbff″0n

### 4.4. Nusselt Number

The Nusselt number is a dimensionless parameter that represents the convective heat-transfer rate at the wall. It is written in the following form:

(24) $$Nu_{x}=\frac{hx}{k_{bf}}$$

Here, h is a convective heat-transfer coefficient that can be written as

(25) $$h=-\frac{k_{nf}\;{\left.\partial_{y}\left(T-T_{\infty }\right)\right|}_{y=0}}{\left(T_{w}-T_{\infty }\right)}$$

After applying transformation Equation (13), it is written as

(26) Nux=−Rex1n+1knfkbfθ′0,

## 5. Solution Technique

The solution of Equations (14) and (15) with respect to Equation (16) is obtained by using the RK method. The method is executed in the following manner:

Let $f=F_{1}$, $\theta =G_{1}$, and convert Equations (14) and (15) into a system of first-order differential equations as

(27) $$\left.\begin{array}{l} F_{1}\prime =F_{2}\\ F_{2}\prime =F_{3}\\ F_{3}\prime =\frac{\frac{\rho_{nf}}{\rho_{bf}}\left[\frac{m}{m+1}F_{2}{}^{2}-\left(\frac{m\left(2n-1\right)+1}{\left(n+1\right)\left(m+1\right)}\right)F_{1}F_{3}\right]-\frac{m}{m+1}}{n\frac{\mu_{nf}}{\mu_{bf}}{\left|F_{3}\right|}^{n-1}}\\ G_{1}\prime =G_{2}\\ G_{2}\prime =-\mathrm{Pr}\frac{k_{bf}}{k_{nf}}\frac{{\left(\rho C_{p}\right)}_{nf}}{{\left(\rho C_{p}\right)}_{bf}}\left(\frac{m\left(2n-1\right)+1}{\left(n+1\right)\left(m+1\right)}\right)F_{1}G_{2} \end{array}\right\}$$

along the initial conditions

(28) $$\left.\begin{array}{l} F_{1}\left(0\right)=0,\\ F_{2}\left(0\right)=-\lambda ,\\ F_{3}\left(0\right)=\Omega_{1}\\ G_{1}\left(0\right)=1\\ G_{2}\left(0\right)=\Omega_{2} \end{array}\right\}$$

Here $\Omega_{1}$ and $\Omega_{2}$ represent unknown boundary conditions. 

To evaluate the accuracy of the results, the values of $f\prime \prime \left(0\right)$ and $-\theta^{\prime }\left(0\right)$ against the parameters of β and Pr are compared with the existing limited results [53,54] in Table 2 and Table 3.

## 6. Result and Discussion

In this segment, the results under the influence of the governing parameters are displayed for discussion. The first set of results is displayed graphically in the form of velocity and temperature profiles related to three homogenous metallic oxide nanofluids under the impacts of different nanoparticle concentrations and diameters. To see the effects of these parameters, the values of the geometry’s parameters, for example, wedge angle Ω=π/6, wedge speed $u_{w}=0.01$, and free-stream velocity $u_{\infty }=0.04$, are taken as fixed, and other parameters, such as the Reynold number and the modified Prandtl number, are varied according to different nanoparticle concentrations and diameters, which are listed in Table 4 and Table 5. It is seen that the Reynold number is varied due to the variation in inertial force. The inertial force is a function of the power-law index, which becomes less than unity when the nanoparticle concentration is increased in the case of the MgO -EG and TiO_2_-EG nanofluids. The modified Prandtl number is a function of the Reynold number and is varied by it.

### 6.1. Velocity Profiles

**Figure 7:**
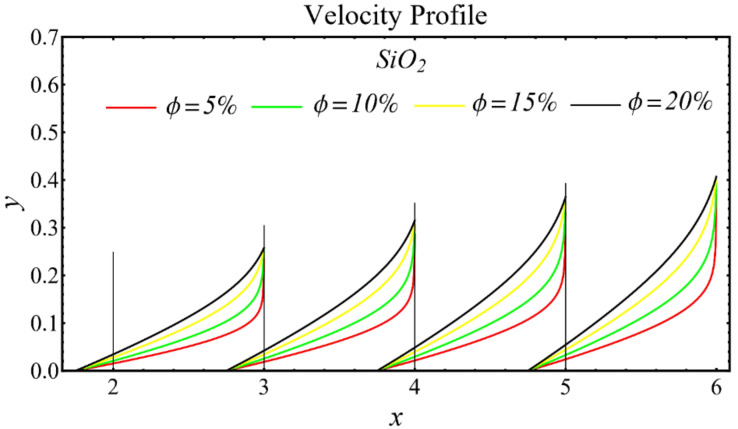
Velocity profile of SiO_2_-EG nanofluid under influence of nanoparticle concentration.

**Figure 8:**
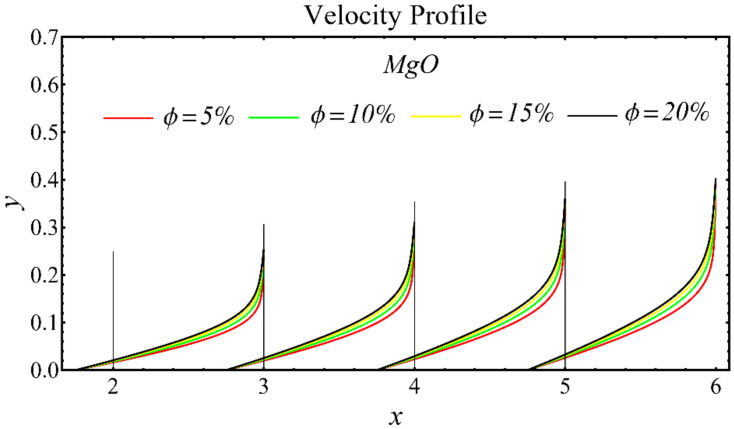
Velocity profile of MgO-EG nanofluid under influence of nanoparticle concentration.

**Figure 9:**
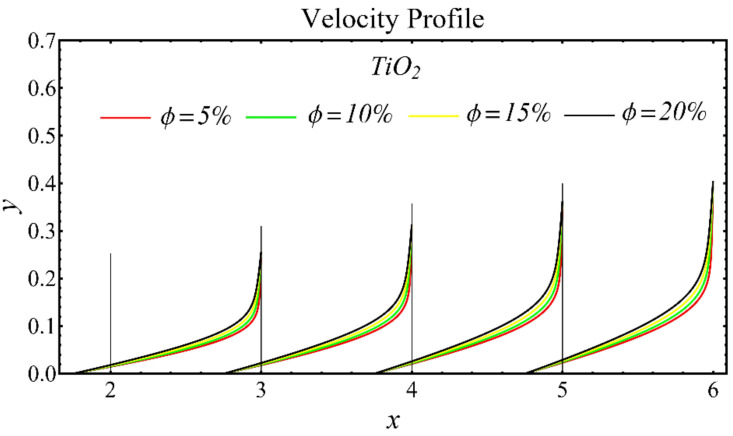
Velocity profile of TiO_2_-EG nanofluid under influence of nanoparticle concentration.

The results of the velocity profiles for the SiO_2_-EG, MgO-EG, and TiO_2_-EG nanofluids under the impact of distinct nanoparticle concentrations are illustrated in Figure 7, Figure 8 and Figure 9. It is seen that the velocity profile for all specified nanofluids is reduced with the increase in nanoparticle concentration. In the current situation, the viscosity of present nanofluids is enhanced due to the increase in nanoparticle concentration, which causes the reduction in velocity. The trend of viscosity and velocity profile for the current study is found to be similar in existing studies [1,55,56,57,58,59,60]. These graphs assert that the dominant effects are found in the velocity profile of the SiO_2_-EG nanofluid due to maximum viscosity as compared with the MgO-EG and TiO_2_-EG nanofluids.

**Figure 10:**
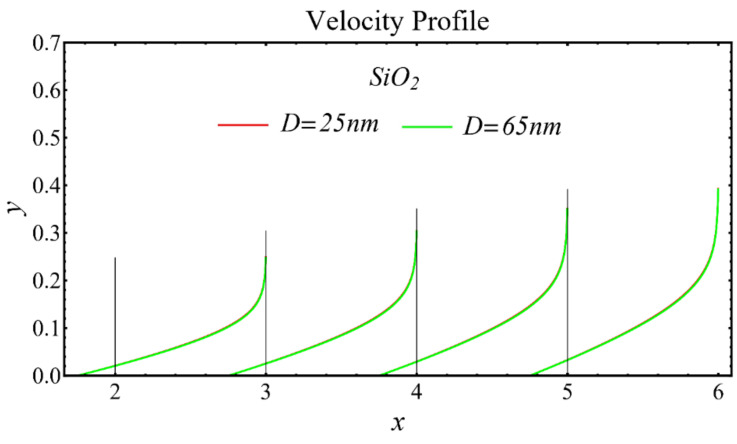
Velocity profile of SiO_2_-EG nanofluid under influence of nanoparticle diameter.

**Figure 11:**
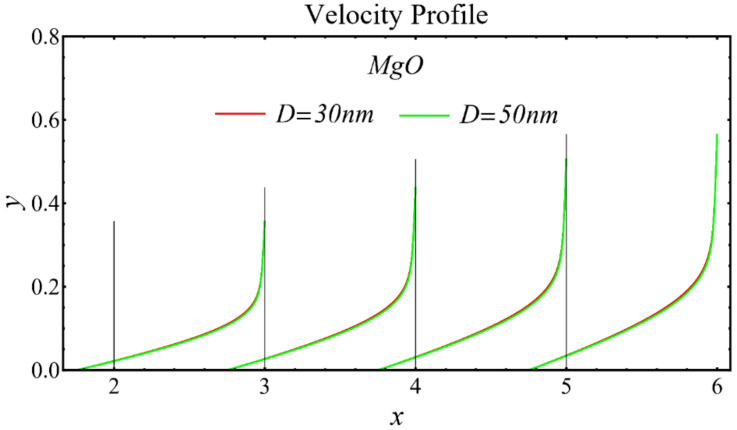
Velocity profile of MgO-EG nanofluid under influence of nanoparticle diameter.

**Figure 12:**
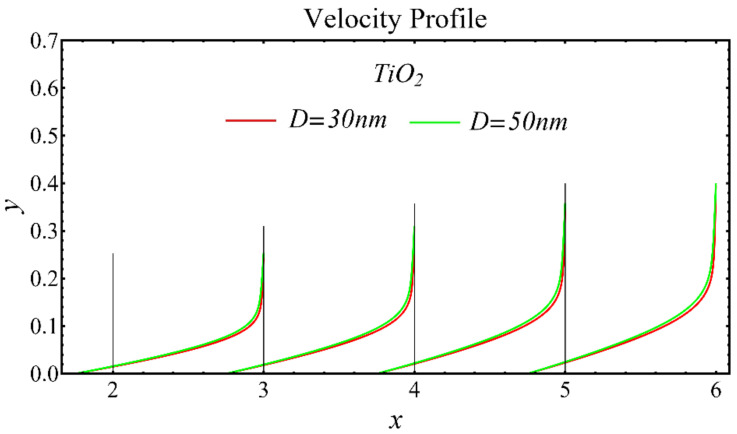
Velocity profile of TiO_2_-EG nanofluid under influence of nanoparticle diameter.

The velocity profiles of the schematic nanofluids under the effect of two distinct nanoparticle diameters are shown in Figure 10, Figure 11 and Figure 12. It is clear that the velocity profile is increased by increasing the nanoparticle diameter due to the decline in the viscosity of the nanofluids. In addition, the dominant impact of this parameter on the profile is found to be more pronounced in the MgO-EG nanofluid than in the other nanofluids.

### 6.2. Temperature Profiles 

**Figure 13:**
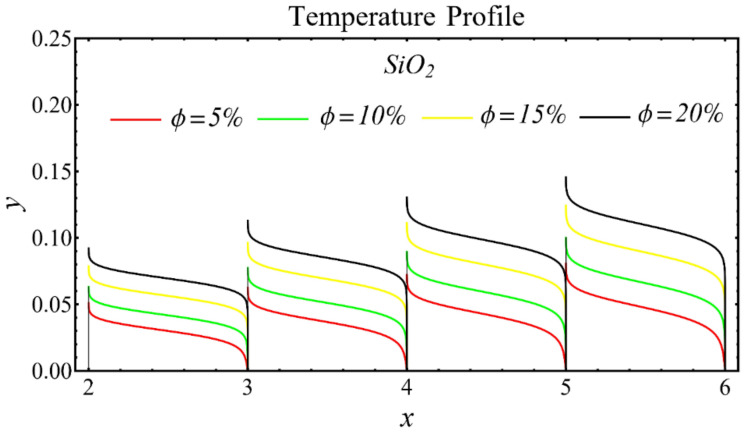
Temperature profile of SiO_2_-EG nanofluid under influence of nanoparticle concentration.

**Figure 14:**
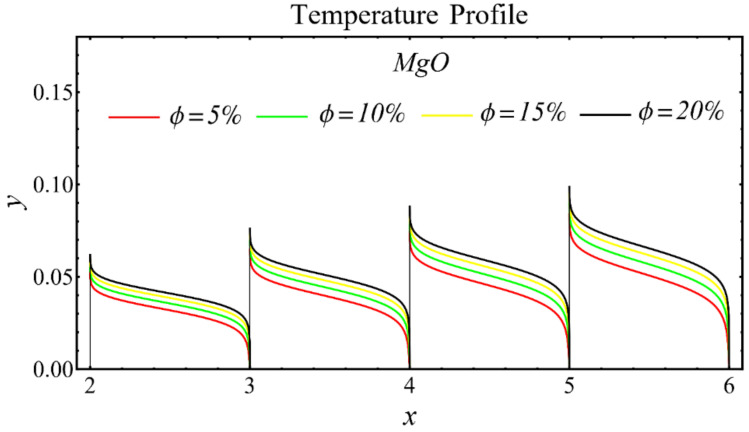
Temperature profile of MgO-EG nanofluid under influence of nanoparticle concentration.

**Figure 15:**
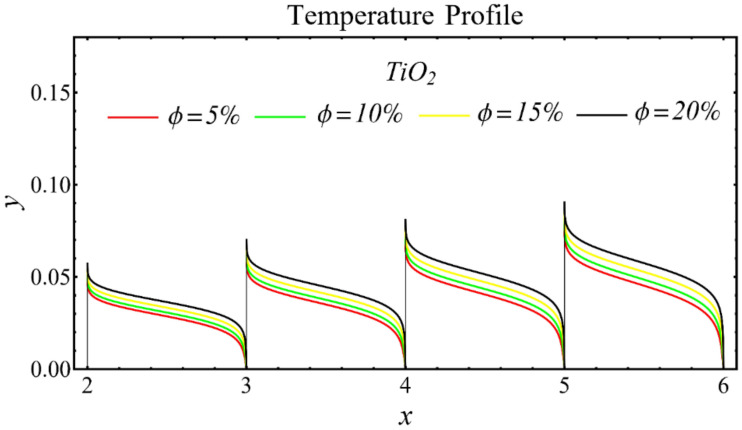
Temperature profile of TiO_2_-EG nanofluid under influence of nanoparticle concentration.

The graphs of the temperature profiles for the nanofluids with respect to various nanoparticle concentrations are displayed in Figure 13, Figure 14 and Figure 15. The figures show that the temperature is raised with the increase in nanoparticle concentration. The fact is that the temperature distribution is enhanced due to the increase in thermal conductivity and the decline in specific heat. The trend of this profile is also matched with the trend demonstrated in existing studies [55,56,57,58,59,60]. Moreover, the graphs disclose that the temperature of the SiO_2_-EG nanofluid is extensively affected by the enhancement of nanoparticle concentrations as compared with the other nanofluids.

**Figure 16:**
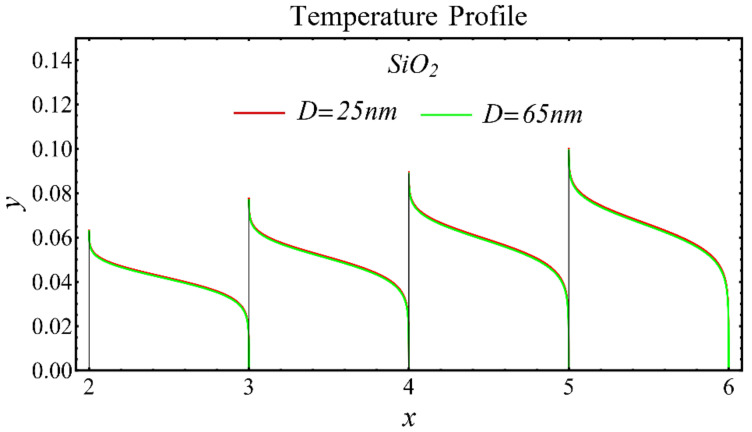
Temperature profile of SiO_2_-EG nanofluid under influence of nanoparticle diameter.

**Figure 17:**
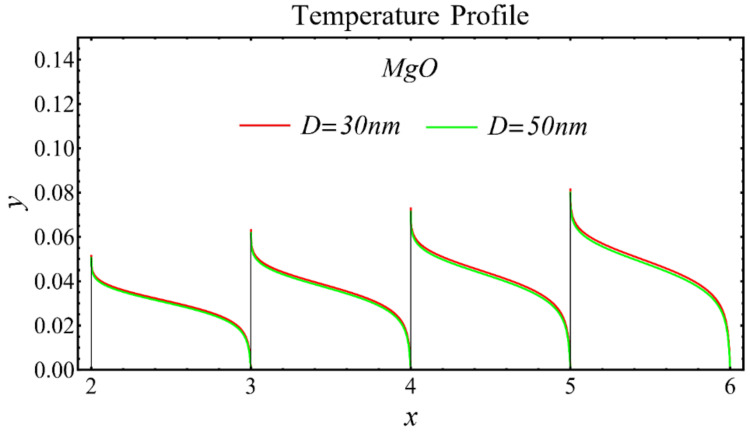
Temperature profile of MgO-EG nanofluid under influence of nanoparticle diameter.

**Figure 18:**
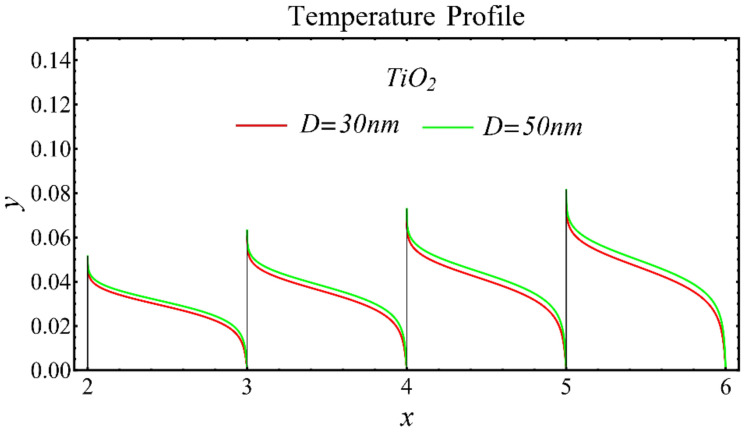
Temperature profile of TiO_2_-EG nanofluid under influence of nanoparticle diameter.

The results of the temperature profiles for the schematic nanofluids under the influence of the nanoparticle diameter are illustrated in Figure 16, Figure 17 and Figure 18. It is revealed that the temperature is reduced by enhancing the nanoparticle’s diameter, as shown in all graphs. This is because of the increase in the Prandtl number that occurs by decreasing thermal diffusion.

### 6.3. Physical Parameters

**Figure 19:**
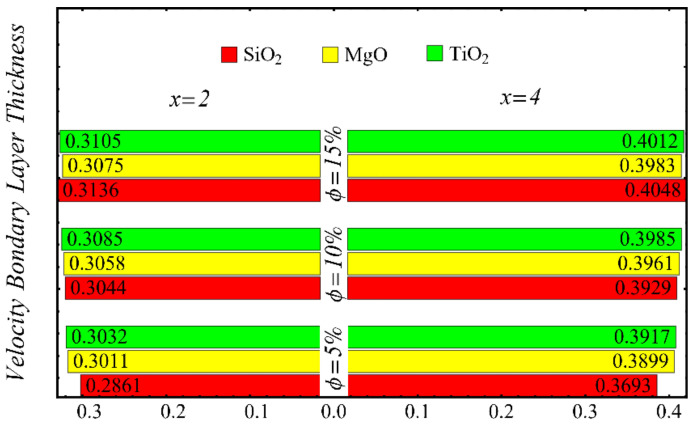
Velocity boundary-layer thickness at different nanoparticle concentrations.

**Figure 20:**
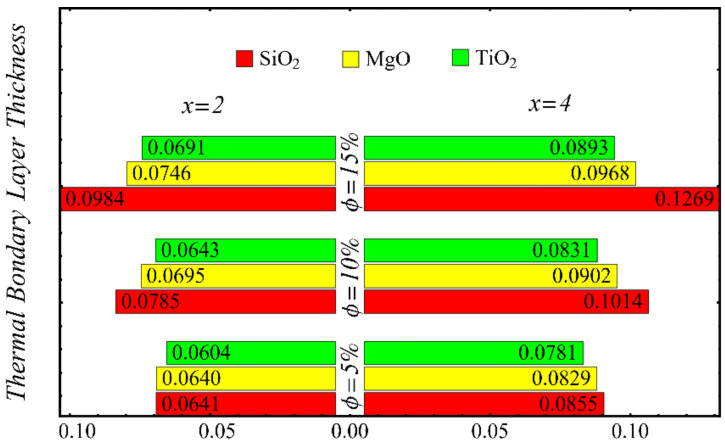
Temperature boundary-layer thickness at different nanoparticle concentrations.

**Figure 21:**
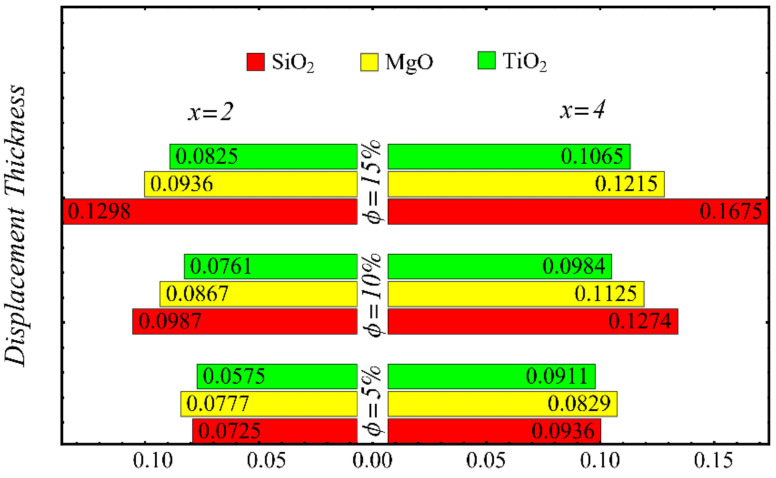
Displacement thickness at different nanoparticle concentrations.

**Figure 22:**
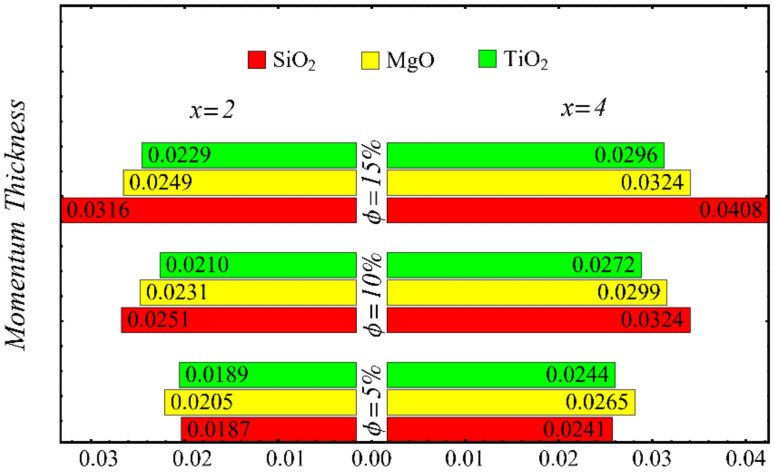
Momentum thickness at different nanoparticle concentrations.

**Figure 23:**
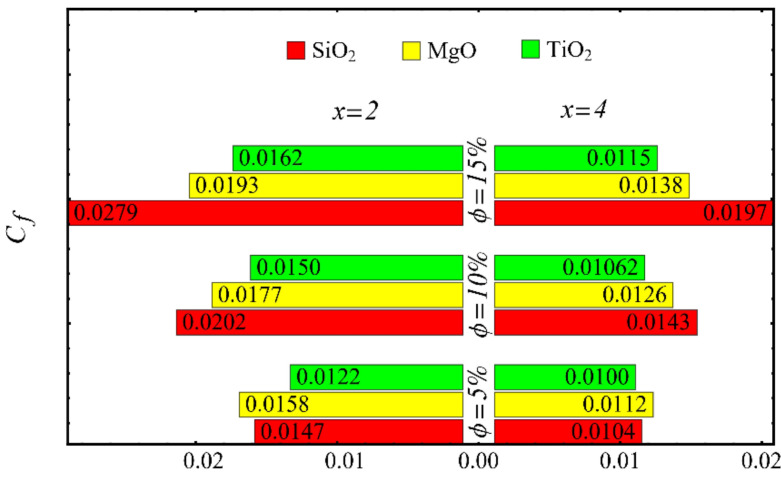
Coefficient of skin friction at different nanoparticle concentrations.

**Figure 24:**
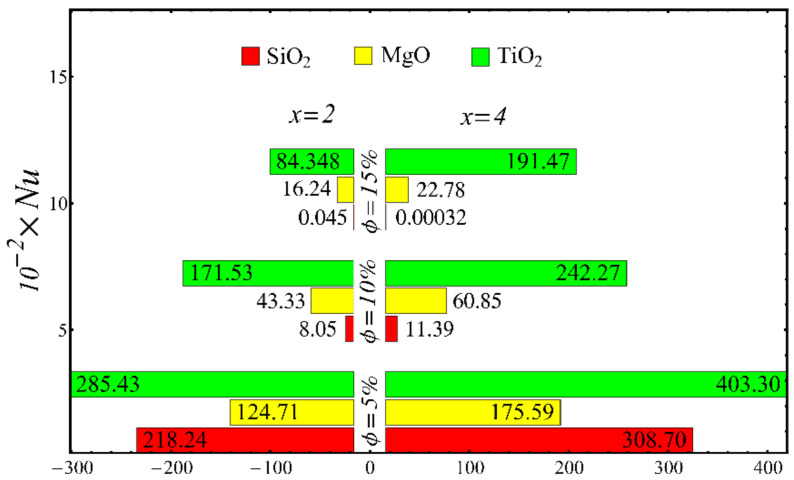
Nusselt number at different nanoparticle concentration.

The results of the boundary-layer parameters, such as the velocity and temperature boundary-region thicknesses, displacement thicknesses, and momentum thicknesses, are presented in Figure 19, Figure 20, Figure 21 and Figure 22, whereas the results of the coefficient of skin friction and the Nusselt number are displayed in Figure 23 and Figure 24.

The velocity and temperature boundary regions’ thicknesses are computed at distinct positions on the surface of the wedge under the impact of nanoparticle concentrations in Figure 19 and Figure 20. It is noted that the thickness of the velocity boundary-layer region is enlarged with the enhancement of the nanoparticle concentration and increases at a position far from the origin. It is also observed that the velocity thickness of the SiO_2_-EG nanofluid is lowest at low concentrations but highest at high concentrations compared with both the MgO-EG and TiO_2_-EG nanofluids. For temperature boundary-layer thickness, it is perceived that the thickness is increased by enhancing the nanoparticle concentration. In addition, the temperature boundary-layer thickness is found to be smaller as compared with the thickness of the velocity boundary layer due to the dominant effects of mass diffusion as compared with thermal diffusion. It is further observed that the thermal boundary-layer thickness is found to be the greatest in the SiO_2_-EG nanofluid as compared with both the TiO_2_-EG and MgO-EG nanofluids.

Figure 21 and Figure 22 show the results of displacement and momentum thicknesses against different nanoparticle concentrations at distinct locations on the wedge’s surface. The value of the displacement thickness is raised by enhancing the nanoparticle concentration and also increases along the distance of the wedge. It is seen that the maximum displacement is found in the case of the SiO_2_-EG nanofluid, and the lowest displacement is found in the TiO_2_-EG nanofluid. The momentum thickness indicates a reduction in the momentum of the nanofluid, and it is observed that the value is increased by raising the nanoparticle concentration.

The values of the coefficient of skin friction for the nanofluids against the nanoparticle concentration are displayed in Figure 23. It is to be noted that the value of the coefficient of skin friction is increased when the nanoparticle concentration is enhanced, and maximum enhancement is found in the case of the SiO_2_-EG nanofluid. The values of the Nusselt number with respect to the nanoparticle concentration are displayed in Figure 24. It is seen that the Nusselt number is decreased with the increase in nanoparticle concentration, and a maximum decline is found in the SiO_2_-EG nanofluid as well. In addition, the trend of the results for the coefficient of skin friction and the Nusselt number agrees with the trend of published studies [55,56,57,58,59,60].

The results of the coefficient of skin friction and the Nusselt number at different values of Rex are shown in Table 6. To calculate the results, the values of Rex in Table 4 are used. The results show the same trend as seen in Figure 23 and Figure 24 because Rex is dependent on the value of the volume fraction.

## 7. Conclusions

In the present analysis, the boundary-layer fluid flow of three homogenous non-Newtonian nanofluids over a moving wedge is investigated. The mathematical results are presented graphically in the form of velocity and temperature profiles and further used to obtain the values of boundary-layer parameters. The main conclusions from the results are as follows:

- The profile of velocity is decreased and increased by raising the values of nanoparticle concentration and diameter, respectively.
- The profile of temperature is increased and decreased by enhancing the values of nanoparticle concentration and diameter, respectively.
- The velocity and temperature boundary-layer regions are increased by increasing the nanoparticle concentration.
- The displacement and momentum thicknesses are increased by the rise of nanoparticle concentration.
- The skin-friction coefficient is enhanced whereas the Nusselt number is decreased with the increase in nanoparticle concentration.

## Figures and Tables

**Table 1:** The values of $A_{1}$, $A_{2}$, $B_{1}$, and $B_{2}$ for different nanofluids at different particle diameters.

	SiO_2_-EG Nanofluid		MgO-EG Nanofluid		TiO_2_-EG Nanofluid	
$d\left(nm\right)\to$	20–30	60–70	20	40	30	50
$A_{1}$	0.025	−0.04	−0.617	−0.0037	1.08	−0.013
$A_{2}$	−0.042	0.104	2.19	−0.112	12.63	−0.17
$B_{1}$	−4.8	−4.2	6.9	1.53	1.08	0.49
$B_{2}$	172.59	160.6	5.02	2.9	12.6	12.2

**Table 2:** Comparison of results for $f^{\prime \prime }\left(0\right)$ with numerical results in [53] when ϕ=0 and n=1.

β	Present	[53]
0	0.46961	0.4696
1	0.92773	0.9277
2	1.23262	1.2326

**Table 3:** Comparison of results for $-\theta^{\prime }\left(0\right)$ with numerical results in [54] when ϕ=0 and β=1.

Pr	Present	[54]
1	0.57052	0.5705
2	0.74370	0.7437
6	1.11471	1.1147

**Table 4:** The values of ${\mathrm{Pr}}_{x}$ and Rex numbers at different nanoparticle concentrations.

		SiO_2_-EG Nanofluid D=20−30nm			MgO-EG Nanofluid D=20nm			TiO_2_-EG Nanofluid D=30nm		
$\frac{\phi \to }{\underset{↓}{x}}$		5%	10%	15%	5%	10%	15%	5%	10%	15%
${\mathrm{Pr}}_{x}$	2	139.5	139.6	139.6	137.9	137.0	136.8	69.7	69.7	138.2
	4	139.5	139.5	139.5	139.1	138.9	138.8	139.4	139.0	278.1
Rex	2	6045	6067	6085	5448	5149	5079	5898	5867	5851
	4	12,098	12,153	12,195	10,705	10,017	9858	11755	11,635	11,667

**Table 5:** The values of ${\mathrm{Pr}}_{x}$ and Rex numbers at different nanoparticle diameters.

		SiO_2_-EG Nanofluid		MgO -EG Nanofluid		TiO_2_-EG Nanofluid	
$\frac{D\left(\mathrm{nm}\right)\to }{\underset{↓}{x}}$		20–30	60–70	30	50	20	40
${\mathrm{Pr}}_{x}$	2	139.6	139.2	137.0	139.4	69.7	139.3
	4	139.5	278.5	138.9	278.7	139.0	278.5
Rex	2	6067	5941	5149	5982	5847	5946
	4	12,153	11,855	10,017	11,951	11,635	11,866

**Table 6:** The values of $C_{f}$ and $Nu_{x}$ at different values of Rex at fixed x=2.

	SiO_2_-EG Nanofluid D=20−30nm			MgO -EG Nanofluid D=20nm			TiO_2_-EG Nanofluid D=30nm		
Rex	6045	6067	6085	5448	5149	5079	5898	5847	5861
$C_{f}$	0.0147	0.0202	0.0279	0.0158	0.0177	0.0193	0.0122	0.0150	0.0162
$Nu_{x}$	218.24	8.05	0.045	124.71	43.33	16.24	285.43	171.53	84.348

## Data Availability

Not applicable.

## Nomenclature

u,v	Velocity components
T	Temperature of fluid
$T_{\infty }$	Temperatures of inviscid region
$u_{\infty }$	Velocity of fluid in inviscid region
k	Thermal conductivity
$C_{p}$	Specific heat
Ω	Wedge angle
τ	Shear stress
$\dot{\gamma }$	Shear rate
ϕ	Volume fraction
x,y	Rectangular coordinates
$T_{w}$	Temperatures of wall
ψ	Stream function
$u_{w}$	Wall velocity
ρ	Density
$\mu_{nf}$	Consistency index
η	Similarity variable
Prx=ρbfcpbfu∞xkbfRex2n−1	Local Prandtl number
Rex=m+1ρxnμu∞n−2	Local Reynold number
**Subscripts**	
nf	Nanofluid
np	Nanoparticle
bf	Base fluid
